# A multi-method spatial examination of factors associated with changes in geographic accessibility to buprenorphine providers in HEALing communities study states Kentucky, Massachusetts, and Ohio

**DOI:** 10.1016/j.pmedr.2025.103045

**Published:** 2025-03-24

**Authors:** Shikhar Shrestha, Olivia Lewis, Daniel Harris, Peter Rock, Anita Silwal, Sumeeta Srinivasan, Thomas J. Stopka

**Affiliations:** a Department of Public Health and Community Medicine, Tufts University School of Medicine, 136 Harrison Ave, Boston, MA 02111, United States of America; b Department of Pharmacy Practice and Science, College of Pharmacy, Lee T. Todd Building, University of Kentucky, Lexington, KY 40506, United States of America; c Institute of Pharmaceutical Outcomes and Policy, College of Pharmacy, Lee T. Todd Building, University of Kentucky, Lexington, KY 40506, United States of America; d Institute for Biomedical Informatics, College of Medicine, University of Kentucky, Lexington, KY 40506, United States of America; e Substance Use Priority Research Area, University of Kentucky, Lexington, KY 40506, United States of America; f School of Community Health Sciences, Counseling, and Counseling Psychology, 441 Willard Hall, Stillwater, OK, 74078, United States of America; g Department of Urban and Environmental Policy and Planning, Tufts University, 503 Boston Ave, Medford, MA 02155, United States of America; h Clinical and Translational Sciences Institute, Tufts University School of Medicine, 35 Kneeland St., Boston, MA 02111, United States of America; i Department of Community Health, Tufts University, 574 Boston Ave, Medford, MA, United States of America

**Keywords:** Buprenorphine, Accessibility, HEALing communities, Spatial analysis

## Abstract

**Objectives:**

Access to substance use treatment is critical to reduce the risk of opioid overdose. However, analyzing geographic accessibility using singular measures may provide imprecise estimates. Our objective was to examine changes in geographic accessibility to buprenorphine providers across three HEALing Communities Study (HCS) states, Kentucky, Massachusetts, and Ohio in the US, using multiple spatial analyses and determining whether disparities in access existed across geographic regions and sociodemographic indicators.

**Methods:**

We used buprenorphine provider data from the Drug Enforcement Administration database in January 2020 (reference point) and June 2022. We used four methods to examine changes in geographic accessibility to buprenorphine: 1) kernel density analysis; 2) change in rates analysis (at the county or municipality level); 3) minkowski distance (drive-time to the nearest provider); 4) enhanced 2-step floating catchment area (E2SFCA) and spatial regression analyses, controlling for HCS communities (Wave 1 or Wave 2) and other sociodemographic factors.

**Results:**

We identified an increase in geographic accessibility to buprenorphine across all three states. Improvements in accessibility were concentrated in areas that had higher reference point access. While our regression model of change in the E2SFCA accessibility index shows that Wave 1 communities in Kentucky had a higher increase in access compared to the state average increase, we were not able to establish consistent associations between HCS communities and changes in the E2SFCA index.

**Conclusion:**

Since geographic accessibility to buprenorphine treatment is critical, additional efforts are needed to improve access to buprenorphine providers in rural areas and areas with limited baseline access.

## Highlights

• Geographic accessibility to buprenorphine increased at all sites (KY, MA, and OH)
• Geographic accessibility increased most in areas with higher baseline access
• Kentucky Wave 1 communities saw greater increases than state average increase
• Rural areas need targeted efforts to improve buprenorphine access

## 1 Introduction

In the US, opioid-related overdose claimed over 80,000 lives in 2022 (CJ et al., 2024) fueled by prescription opioids, heroin, and, in more recent years, the involvement of synthetic opioids like fentanyl, as well as stimulants and poly-substance use (Ciccarone, 2021; Ciccarone and Shoptaw, 2022). The concerning trend has been further complicated by the ever-changing drug supply, challenges in the provision of medications for opioid use disorder (MOUD), and stigma (Ciccarone, 2017; Mitchell et al., 2022; Cernasev et al., 2021; Abraham et al., 2022; Morgan et al., 2022). While MOUD (e.g., methadone, buprenorphine) (Degenhardt et al., 2023) are evidence-based treatment options that help reduce opioid-related overdose risk (Larochelle et al., 2018), access to MOUD is limited (Mitchell et al., 2022; Amiri et al., 2021a; Amiri et al., 2021b; Hirchak et al., 2022).

The Comprehensive Addiction and Recovery Act of 2016 (CARA, P.L. 114–198) relaxed training policies and expanded the types of clinicians who can prescribe buprenorphine (e.g., nurse practitioners and physician assistants who could prescribe after 24 h of specialized training) and increased prescription limits (from 30 to 100 patients after the first year, and up to 275 patients after the second year if they meet additional criteria), allowing for greater availability of buprenorphine. Between 2017 and 2020, the number of potential treatment slots grew from 1.75 to 5.12 million nationwide (Andrilla and Patterson, 2022). Expansion of access during the SARS-CoV-2 (COVID-19) pandemic through waivers and the use of telemedicine created additional opportunities for people with opioid use disorder to access buprenorphine (Medicare Cf, Services M, 2020). On December 29, 2022, the Mainstreaming Addiction Treatment (MAT) Act was passed, removing the X-waiver requirement to reduce barriers to buprenorphine prescribing and treatment (Roy et al., 2024; Office of the Federal Register National Archives and Records Administration, 2022). Despite these changes, there are still vulnerable populations who do not have adequate access to buprenorphine (Mitchell et al., 2022; Amiri et al., 2021a; Amiri et al., 2021b; Saloner et al., 2022; Jones, 2018). Geographical and structural barriers remain as a major cause for concern; rural residents have reported longer travel times to buprenorphine providers (Amiri et al., 2021a; Amiri et al., 2021b), hospitals and health centers in rural areas had limited provisions of buprenorphine (Jones, 2018; Pham et al., 2022; Bond Edmond et al., 2015), and physicians in rural areas reported higher levels of biases that might limit access (Franz et al., 2021). In addition, several studies report barriers to buprenorphine distribution by pharmacies as they could be subjected to excessive Drug Enforcement Administration (DEA) oversight, leading them to limit orders and decline prescriptions (Kazerouni et al., 2021; Textor et al., 2022; Qato et al., 2022). Disparities and barriers to treatment access are also driven by income (McClellan et al., 2019), race-ethnicity (Hirchak et al., 2022; Schuler et al., 2021), and, in rural communities, many other socioecological factors (Stopka et al., 2024a). However, with increasing access to MOUD treatment, some studies report that distance to providers may not be a barrier in some communities (Davidson et al., 2022), nonetheless, rural communities still have limited access to both methadone and buprenorphine.

The HEALing (Helping to End Addiction Long-term^SM^) Communities Study (HCS) is a four-year, multisite, parallel-group, cluster-randomized, wait-list controlled trial testing community-level interventions that are aimed at reducing opioid-related overdoses in 67 communities across Kentucky (KY), Massachusetts (MA), Ohio (OH), and New York (NY) in US by implementing community-driven strategies to adopt evidence-based practices (HEALing Communities Study Consortium, 2020). Intervention options and characteristics, including interventions aimed at increasing buprenorphine treatment initiation and continuation, are described elsewhere (Stopka et al., 2024b).

The objective of our study was to examine changes in geographic accessibility to buprenorphine providers across three HCS states, KY, MA, and OH (NY was not included due to data unavailability) using multiple spatial analyses and to determine whether disparities in access existed across geographic regions and sociodemographic indicators. We hypothesized that the increase in accessibility would be higher in HCS intervention communities (Wave 1) compared to waitlist-control (Wave 2) or non-HCS communities.

## 2 Methods

Measurement of geographic accessibility to services is commonly conducted at a prespecified administrative unit of analysis (such as census tracts, zip codes, or counties) or, in more recent years, using gravity-based methods such as floating catchment area indices (Luo, 2004; Luo and Qi, 2009; Shrestha et al., 2023). However, these analyses have several limitations, including challenges in interpretability, inconsistent boundary definition, scale and resolution issues, modifiable areal unit problems, and aggregation bias. Limiting an analysis to a singular spatial unit may introduce bias in GIS analysis, therefore, we used a multi-pronged approach by assessing changes in access using varying spatial units and methods. We assessed the change in geographic accessibility to buprenorphine providers between the start (henceforth referred to as *reference point*, January 2020) and end of the Wave 1 intervention period (June 2022), which approximately reflects the period when intervention communities received Communities that Heal (CTH) intervention (January 1, 2020 – June 30, 2022) across the HCS states. We used four different methods to examine the change in geographic accessibility to buprenorphine providers: i) calculating the change in density of buprenorphine providers using raster analysis, ii) calculating the change in rates of buprenorphine providers per 100,000 people at the county or municipality level, iii) examining the change in geographic accessibility to buprenorphine providers as a function of driving time to the closest buprenorphine provider using Minkowski distance (a generalized metric for measuring the distance between two points) as proxy, and iv) examining differences in accessibility to buprenorphine providers by using an enhanced 2-step floating catchment area method (E2SFCA) while controlling for HCS communities (Wave 1 or Wave 2) and other sociodemographic indicators through multivariable regression modeling.

### 2.1 Data

We used data from the U.S. DEA to identify buprenorphine providers (Supplemental Table 1) who had received Drug Addiction Treatment Act (DATA) of 2000 waivers (X-waiver). After geocoding, we aggregated buprenorphine providers at the census tract level in all HCS states (KY, MA, and OH) included in the analysis. Buprenorphine providers practicing at the same location were aggregated when applicable for the analyses.

We compiled the following sociodemographic data from the U.S. Census Bureau's American Community Survey (ACS) 5-year estimates at the census tract level for the years 2016–2020: percent (%) of the population over the age of 18 years, population density (people per square mile), % non-Hispanic White population, % non-Hispanic Black population, % non-Hispanic Asian population, % Hispanic population, % married household, % of people aged 25 or over with a high-school diploma or higher level of education, % unemployed for people aged 16 years or older, median household income, Gini index, % vacant housing units, % of people without health insurance, and % of people with private health insurance.

We obtained county-level and census tract-level shapefiles for the analysis from the U.S. Census Bureau**.** For MA, we obtained the municipality-level shapefile from MassGIS, Massachusetts's Bureau of Geographic Information (MassGIS, 2025).

### 2.2 Spatial and statistical analyses

*Kernel density analyses:* We used kernel density analysis and analyzed differences in densities to examine the change in the number of buprenorphine providers. We first calculated kernel density estimates between January 2020 and June 2022. We used a cell size of 1000 and a search radius of 25,000 m for KY and OH, and 6500 m for MA using a modified form of Scott's rule (Scott, 2015). We then used the raster algebra tool in ArcGIS Pro to calculate differences in the density values. We examined changes in the density of buprenorphine providers on aggregate across all counties/municipalities.

*Change in rate analyses:* We calculated rates of buprenorphine providers per 100,000 people at the county level for KY (120 counties) and OH (88 counties), and at the municipality level for MA (351 municipalities) between January 2020 and June 2022. We then calculated the percent change in rates of buprenorphine providers across the study period to examine changes in geographic accessibility to buprenorphine providers at the county/municipality level.

*Minkowski distance analyses:* Drive-time analysis can provide a quantifiable and easily understandable measure of geographic access to services, but these estimates can be computationally challenging to obtain at a large scale. Minkowski distance is a metric used to measure the distance between two points in a normed vector space, generalizing both the Euclidean distance (when *p* = 2) and the Manhattan distance (when *p* = 1).

$$D\left(X,Y\right)={\left(\sum_{i-1}^{n-1}{\left|x_{i}-y_{i}\right|}^{p}\right)}^{\frac{1}{p}}$$

where x (x_1_, x_2_, …. x_n_) and y (y_1_, y_2_, …, y_n_) are two points in n-dimensional space, and p is power which determines the type of distance.

By employing Minkowski distance, we achieve a consistent and computationally feasible means of comparing geographic accessibility to buprenorphine providers across diverse locations. Shahid et al. report that a *p*-value of 1.31 best approximates drive-time between two locations (calculated with the assumption of average speed of 60 km/h ∼ 37.3 miles/h) (Shahid et al., 2009). We calculated Minkowski distance using the population-weighted centroids of census tracts as demand points and the location of the closest buprenorphine provider as the supply point. We then converted the distance into time in minutes, assuming a driving speed of 37.3 miles/h (60 km/h). Finally, we graphed the cumulative percentage of the population at the census tract-level on the y-axis and drive-time on the x-axis to assess geographic accessibility to buprenorphine providers sorted by drive-time.

*Enhanced 2-step floating catchment area and spatial regression analyses*: We conducted E2SFCA analyses to assess geographic accessibility to buprenorphine providers across HCS states. An enhanced floating catchment area index (Luo, 2004; Luo and Qi, 2009; Chen and Jia, 2019) considers both the supply and demand aspect of geographic accessibility. The detailed methods for estimating E2SFCA are described elsewhere (Luo and Qi, 2009; Shrestha et al., 2023). We used the model parameters listed below for our study:

1) Supply locations: buprenorphine providers along with buprenorphine prescribing capacity.
2) Demand locations: population-weighted centroids at the census tract level, estimating the population in need of buprenorphine treatment, from ACS 5-year estimates (2016–2020).
3) Distance parameter: Drive-time – determined by state average commute time (U.S. Census Bureau, n.d.)

After calculating these indices, we employed a multivariable regression model to examine the changes in geographic accessibility to buprenorphine providers between the study time points using reference point accessibility and other predictors. To obtain the final set of predictors, we started with a broad range of predictors (mentioned in section 2.1) (Mitchell et al., 2022; Shrestha et al., 2023; Mitchell et al., 2023; Kolak et al., 2020). HCS community indicator as a variable was chosen a priori. We then identified a set of covariates for inclusion in the final model based on bivariate regression (significant at *p* < 0.2) and removed variables that showed a high degree of multicollinearity. The final set of covariates included HCS community indicator (Wave 1, Wave 2, or non-HCS community), population density (also a proxy for urbanicity), percentage of non-Hispanic white population, median household income, Gini index, and population without health insurance as predictors.

We used ArcGIS Pro (Version 3.2, Redlands, California) for spatial analysis and Python (version 3.11) for statistical analysis. This study protocol (Pro00038088) was approved by Advarra Inc., the HEALing Communities Study Single Institutional Review Board.

## 3 Results

**Fig. 1:**
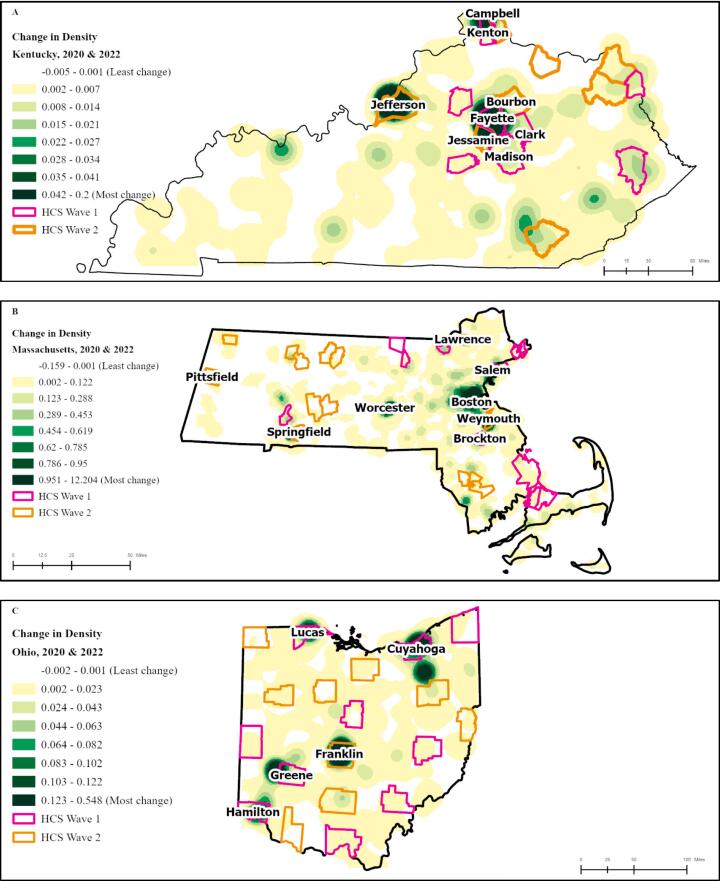
Change in Density of Buprenorphine Providers Between January 2020 and June 2022 in A. Kentucky B. Massachusetts, and C. Ohio.

*Change in density of buprenorphine providers between 2020 and 2022*: In KY, OH, and MA, the areas with the highest increase in density of buprenorphine providers between 2020 and 2022 were urban centers (Fig. 1). In KY, the cities of Louisville, Lexington, Florence, and Covington had the highest increases in density of buprenorphine providers; these cities are all located in or next to HCS communities (Wave 1 communities Kenton, Fayette, Madison, and Clark Counties, and Wave 2 communities Jefferson, Jessamine, and Bourbon Counties). In MA, we observed the highest density increases in Boston, Worcester, and Springfield; Springfield is a Wave 2 community. The Wave 2 communities Pittsfield, Weymouth, and Lawrence, as well as Wave 1 communities Brockton and Salem, also had high increases in density during the study period. Similarly, in OH, we observed the highest increases in density in Columbus, Cleveland, and Cincinnati, as well as Dayton, Toledo, and Akron. Almost all these cities were located within or adjacent to Wave 1 communities, except for Columbus, which was located in Franklin County, a Wave 2 community.

**Fig. 2:**
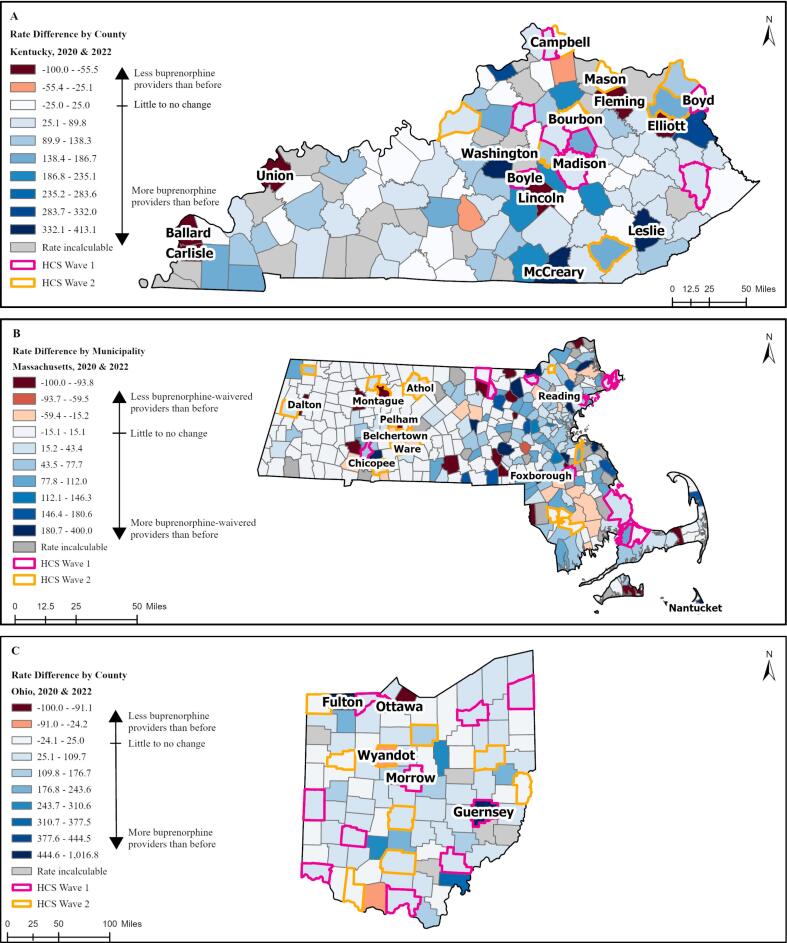
Difference in Rates of Buprenorphine Providers per 100,000 Population Between January 2020 and June 2022 in A). Kentucky, B). Massachusetts, and C). Ohio.

*Change in rates of buprenorphine providers between 2020 and 2022*: We found that in most counties or municipalities (82 % of counties in KY, 90.3 % of counties in OH, and 44.8 % of municipalities in MA) across all states, the rates of buprenorphine providers increased (Fig. 2). Most HCS Wave 1 and Wave 2 communities in KY experienced small increases in the rates of buprenorphine providers, except for Boyle, Boyd, Bourbon, Campbell, and Mason Counties, which saw little to no change.

A large proportion of the municipalities in MA (43 %) showed no change, and approximately 100 cities or towns did not have any buprenorphine providers at either timepoint (Fig. 2). All HCS Wave 2 communities in MA had small to moderate increases in rates of buprenorphine providers, HCS Wave 1 communities had mixed results; rates of providers decreased in Athol, Belchertown, Montague, and Ware while there were moderate increases in all other Wave 1 communities.

In OH, the largest decrease in the rate of buprenorphine providers was in Ottawa County in northern Ohio, while Fulton and Guernsey Counties (northern and southeastern Ohio respectively) had the highest rate increases (Fig. 2). Most HCS Wave 1 communities in OH had modest increases in provider rates, except for Guernsey, Morrow, and Wyandot counties. HCS Wave 2 communities in Ohio had little to no change in provider rates (Fig. 2).

**Fig. 3:**
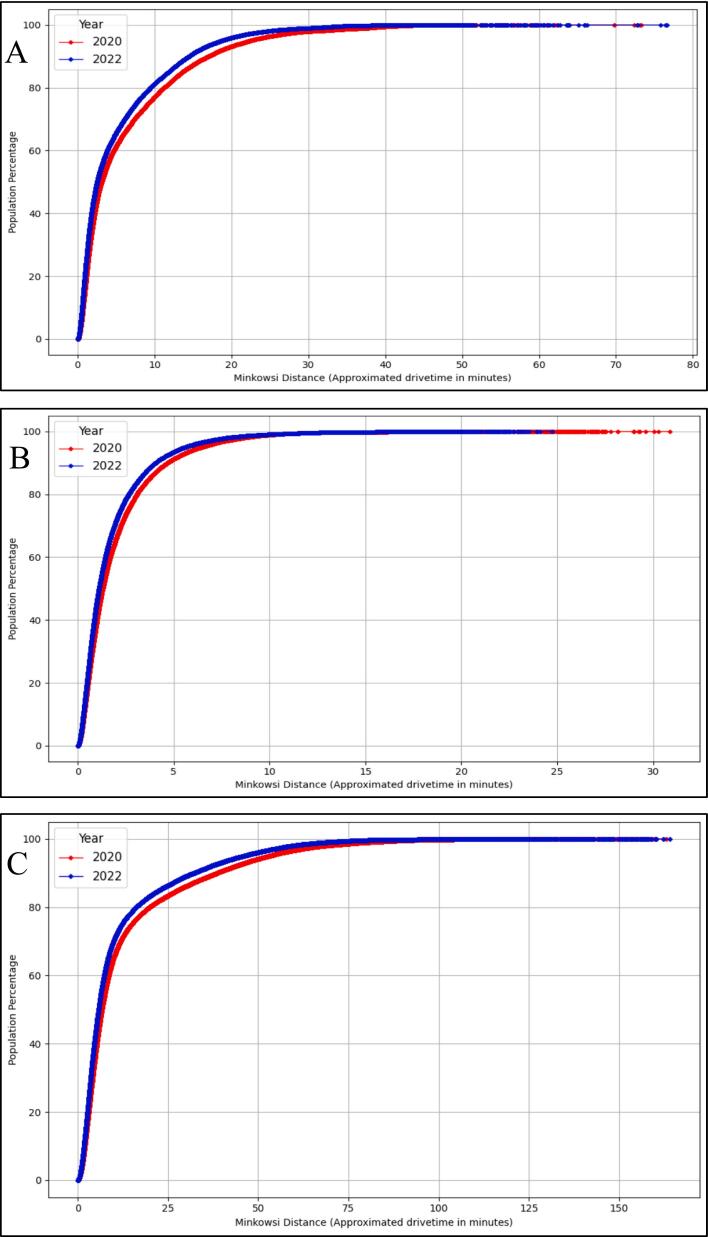
Difference in Minkowski Distance (*p* = 1.31) to Approximate Drive-Time Between January 2020 and June 2022 in A. Kentucky, B. Massachusetts, and *C. Ohio*.

Change in Minkowski distance to closest buprenorphine providers from population-weighted centroids of census tracts: Across all three states, we found that the Minkowski distance-based drive-time approximation to the closest buprenorphine provider decreased between 2020 and 2022 (Fig. 3). In KY, the proportion of people living within 10-min drive of a buprenorphine provider increased from 75 % to 82 % between 2020 and 2022. In 2020, approximately 90 % of the MA population lived within a 6-min drive of a buprenorphine provider, and by 2022, the proportion had increased to over 92 %. In OH, approximately 88 % of the population lived within a 10-min drive of the closest buprenorphine provider in 2020, and by 2022 the proportion had increased to approximately 90 %.

**Fig. 4:**
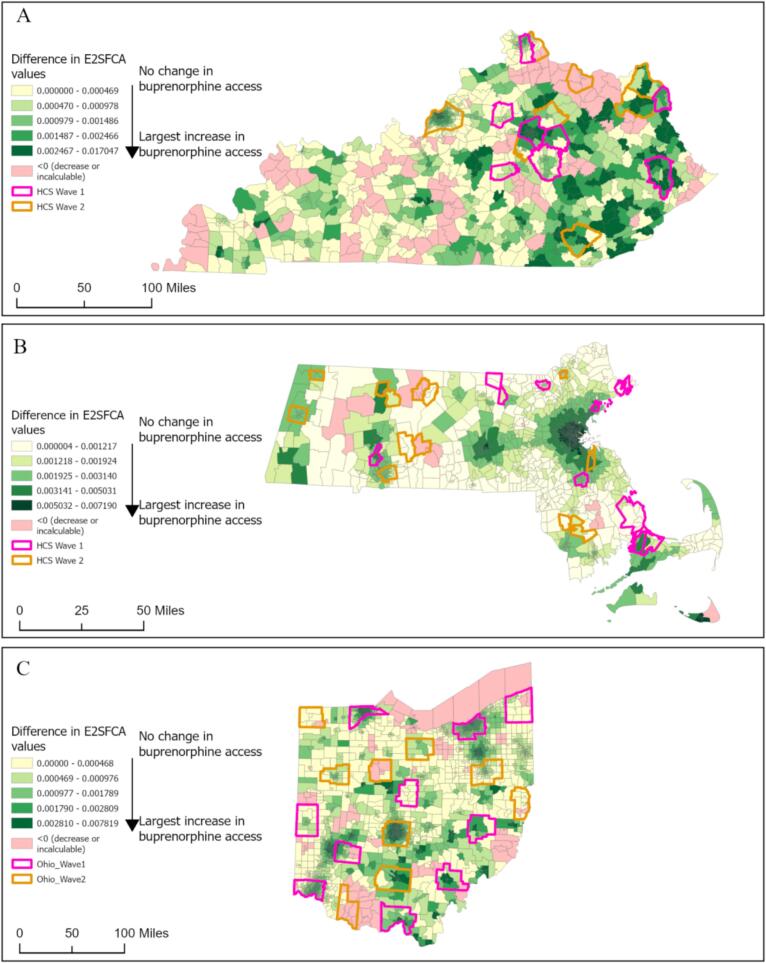
Differences in Enhanced 2-Step Floating Catchment Area Accessibility (E2SFCA) Index Between Years 2020 and 2022 in A. Kentucky, B. Massachusetts, and *C. Ohio*.

*Factors associated with changes in E2SFCA accessibility index*: In most of the census tracts across all three states, the E2SFCA (difference in E2SFCA between 2020 and 2022) index increased (Fig. 4), signifying improvement in geographic accessibility. Our regression model showed that the E2SFCA index at the reference point was associated with statistically significant increases in the final E2SFCA index in all three states (KY: 0.55, MA: 0.76, OH: 0.61, all statistically significant, Table 1). In KY, the change in E2SFCA indices among Wave 1 communities was 0.258 more than the mean change in the state, whereas, in MA and OH, the change in the E2SFCA indices was 0.19, and 0.06, less than the mean change in the E2SFCA indices for the entire states. Additionally, for census tracts within Wave 2 communities in MA, the change in the E2SFCA index was 0.62 less than the overall mean change in the state; for Ohio, the change was higher than the state average by 0.25; however, for KY Wave 2 communities, the change was not statistically significant (Table 1). Population density had a significant impact on increases in E2SFCA indices across all states (KY: 0.006, MA: 0.01, and OH: 0.01). We did not see consistent patterns when examining the impact of other socio-economic covariates on change in E2SFCA scores across all three states.

**Table 1:** Independent associations between changes in accessibility indices (outcome) and reference point accessibility, HCS communities, and sociodemographic indicators for Kentucky, Ohio, and Massachusetts, Jan 2020 – June 2022.

	Kentucky			Massachusetts			Ohio	
	Beta Coefficient	95 %CI		Beta Coefficient	95 %CI		Beta Coefficient	95 %CI
Reference point E2SFCA Index (Jan 2020) (Standardized)	0.552	(0.505–0.598)		0.7584	(0.732–0.785)		0.6136	(0.582–0.646)
Wave 1 Communities	0.258	(0.129–0.388)		−0.1953	(−0.273- -0.118)		−0.0639	(−0.126- -0.002)
Wave 2 Communities	0.021	(−0.106–0.148)		−0.617	(−0.696- -0.537)		0.2499	(0.177–0.323)
Non-Hispanic White Percent	−0.002	(−0.005–0.001)		−0.0049	(−0.006- -0.004)		−0.001	(−0.002–0)
Median Household Income (Standardized)	−0.003	(−0.006–0.001)		0.0066	(0.005–0.008)		0.0015	(−0.001–0.004)
Gini Index (Standardized)	0.003	(−0.002–0.007)		0.0032	(0.001–0.005)		−0.0001	(−0.002–0.002)
Population Density (Standardized)	0.006	(0.003–0.01)		0.0107	(0.009–0.013)		0.014	(0.011–0.017)
Uninsured Percent	0.001	(−0.003–0.004)		−0.0038	(−0.013–0.005)		0.0068	(0.002–0.011)

Footnotes: CI: Confidence Interval, E2SFCA: Enhanced 2-step floating catchment area, Wave 1: Healing Communities study intervention communities, Wave 2: Healing Communities study waitlist control communities, Gini Index: Index of economic inequality derived from American Community Survey Data.

## 4 Discussion

Examining geographic access to buprenorphine providers requires a nuanced approach to mitigate biases inherent in selecting specific units of analysis. To address this, we employed a multi-spatial unit, multi-model examination, utilizing four distinct methods. These included assessing raster differences without spatial aggregation, rate differences at county/municipality levels, Minkowski distances, and changes in geographic accessibility controlling for HCS communities and socioeconomic indicators. By adopting this comprehensive approach, we provide a robust assessment of geographic access, capturing variations across different spatial scales and methodologies. Our findings indicate an overall increase in geographic access to buprenorphine providers across all three states, suggesting potential improvements in availability and distribution. We did not observe a consistent association between being a Wave 1 or Wave 2 HCS community and change in access, highlighting the complex interplay of factors influencing the availability of buprenorphine providers. As expected, geographic accessibility at the start of Wave 1 was the strongest predictor of a greater increase in accessibility during the intervention period, meaning that areas with higher levels of access at the beginning of the study also had the highest increase at the end of the Wave 1 period.

Across all three study states, we observed increased geographic accessibility to buprenorphine providers. Our findings are consistent with reports highlighting an increase in the number of buprenorphine providers as every year, more providers obtained data waivers (until the new laws nullifying the X-waiver were passed) (Roy et al., 2024; Luo and Erikson, 2023). A multitude of efforts were implemented to increase buprenorphine prescribing by providers. Practice Guidelines for Administration of Buprenorphine for Treating Opioid Use Disorder (Health UDo, Services H, 2021) were issued in early 2021, and a study reported that the guidelines led to a 16 % increase in buprenorphine providers by the end of the year (Health UDo, Services H, 2022). While efforts to incentivize providers to get waivers and prescribe have been largely successful, studies report that there has not been any significant changes in buprenorphine prescribing rates and that a small number of buprenorphine providers actually prescribed buprenorphine (Luo and Erikson, 2023; Savitz et al., 2024; Jones et al., 2023).

Overall, our findings are congruent with a report highlighting that the CTH Intervention was not associated with a consistent increase in buprenorphine providers (Stopka et al., 2024b). While the number of prescribers increased in both Wave 1 and Wave 2 communities, the overall increase in geographic accessibility to buprenorphine providers in most HCS communities was no larger than the average increase in geographic accessibility to buprenorphine providers in the study period. We previously analyzed geographic accessibility to buprenorphine in HCS communities using E2SFCA accessibility indices to identify areas with low buprenorphine access, which was shared with communities for planning purposes (Shrestha et al., 2023). It is important to note that our current findings using two-time points are largely consistent with our prior work, which was limited to a single point in time (March 2021). Our study is also in agreement with other published reports, and shows significant increases in access to buprenorphine providers, the increases are primarily occurring in urban areas with higher reference point geographic accessibility. People who use drugs (PWUD) in rural areas consistently have limited access or have to travel longer distances to reach both buprenorphine or methadone (Amiri et al., 2021a; Amiri et al., 2021b; Hirchak et al., 2022; Andrilla and Patterson, 2022). This highlights a significant challenge as rural patients are equally impacted by opioid overdose compared to urban areas but lag in access to treatment (Amiri et al., 2021a; Amiri et al., 2021b; SM, 2021). Furthermore, the increasing rates of buprenorphine providers have not been correlated with increases in buprenorphine prescriptions, indicating that there are factors beyond availability and accessibility that may be affecting treatment uptake (Stopka et al., 2024b; Luo and Erikson, 2023; Savitz et al., 2024). The number of providers is only one component (supply) of measuring accessibility; the population (demand) and drive-time (distance) also drive the calculation, and as long as the population or demand remains fixed, we may improve access by reducing the drive-time burden. Travel vouchers to ease the burden of treatment distance, providing free rides to treatment, may be effective, given that 9 % of the U.S. population lives outside a 10-mile radius of the closest buprenorphine treatment provider, and many people seeking treatment do not have access to a car or other reliable transportation (Langabeer et al., 2020).

Challenges to buprenorphine dispensing by pharmacies are also an additional concern (Kazerouni et al., 2021; Textor et al., 2022; Qato et al., 2022). Some pharmacists noted distance to pharmacist or provider as a “red flag,” which could impact dispensing legally prescribed buprenorphine to patients (Textor et al., 2022). Additionally, pharmacies in rural areas and in southern states were less likely to stock or dispense buprenorphine (Kazerouni et al., 2021; Welsh et al., 2003). Future studies should examine how distance to pharmacies and prescribers impacts initiation and adherence to buprenorphine treatment.

Despite the strengths of our study in using multiple spatial scales and models to understand the change in buprenorphine access, several limitations should be acknowledged. We used the location of the buprenorphine provider as a proxy for access to buprenorphine prescriptions as we lacked granular spatial data. Additionally, the presence of a provider does not equal the actual prescription or treatment. Still, such location data are routinely used to analyze access in several healthcare services research. Our team recently published a study highlighting null findings on the effects of HCS intervention regarding X-waiver receipt and buprenorphine prescribing among providers with a waiver (Stopka et al., 2024b). Although the waiver requirement has been removed, there has still been no change in buprenorphine prescription patterns in HCS states (Christine et al., 2024), pointing to the need for extensive training and buprenorphine prescription promotion for providers. The reliance on aggregated count and rate data may obscure finer spatial patterns of geographic accessibility, and the absence of real-time data limits the temporal dimension of our analysis. However, we mitigate some of these limitations using aggregated (municipality/county, census tract) and non-aggregated (raster) measures. We also did not consider overall urban-rural status across all states but relied on population density, which correlates well with urban-rural status. In the Minkowski distance analysis, we used 37 miles per hour (60 km/h) as the average driving speed to perform the drive-time calculation – this may vary significantly across states and urbanicity. Additionally, while our multi-methods approach offers robustness to variation in spatial scale, each methodological approach has its own assumptions and limitations. For our multivariable models, we did not account for spatial lagged effects of the geographic accessibility at the reference point or the underlying socio-economic features on the change in accessibility. Additionally, in our E2SFCA analysis, we used population above the age of 18 to estimate the demand for buprenorphine treatment rather than number of people with opioid use disorder.

Our findings show that across the three states (KY, MA, and OH included in this analysis) participating in the HCS, geographic accessibility to buprenorphine providers increased; however, the changes in buprenorphine provider access in HCS communities (both Wave 1 and Wave 2) were not significantly different from the average change in access across the states. We found that the primary drivers of increased geographic accessibility were reference point values of geographic access and population density. Our findings show that additional interventions are needed to improve geographic accessibility among PWUD in rural areas.

## CRediT authorship contribution statement

**Shikhar Shrestha:** Writing – review & editing, Writing – original draft, Visualization, Supervision, Methodology, Formal analysis, Conceptualization. **Olivia Lewis:** Writing – review & editing, Writing – original draft, Visualization, Software, Methodology, Formal analysis. **Daniel Harris:** Writing – review & editing, Methodology, Data curation, Conceptualization. **Peter Rock:** Writing – review & editing, Data curation. **Anita Silwal:** Writing – review & editing. **Sumeeta Srinivasan:** Writing – review & editing, Methodology, Conceptualization. **Thomas J. Stopka:** Writing – review & editing, Supervision, Investigation, Conceptualization.

## Funding sources

“This research was supported by the National Institutes of Health and the Substance Abuse and Mental Health Services Administration through the NIH HEAL (Helping to End Addiction Long-term®) Initiative under award numbers UM1DA049394, UM1DA049406, UM1DA049412, UM1DA049415, UM1DA049417 (ClinicalTrials.gov Identifier: NCT04111939). This study protocol (Pro00038088) was approved by Advarra Inc., the HEALing Communities Study single Institutional Review Board. We wish to acknowledge the participation of the HEALing Communities Study communities, community coalitions, community partner organizations and agencies, and Community Advisory Boards and state government officials who partnered with us on this study. The content is solely the responsibility of the authors and does not necessarily represent the official views of the National Institutes of Health, the Substance Abuse and Mental Health Services Administration or the NIH HEAL Initiative®.”

## Declaration of competing interest

The authors declare that they have no known competing financial interests or personal relationships that could have appeared to influence the work reported in this paper.

## Data Availability

The authors do not have permission to share data.

## Appendix A Supplementary data

Supplementary material Image 1
