# Supplementary material for: A multi-method spatial examination of factors associated with changes in geographic accessibility to buprenorphine providers in HEALing communities study states Kentucky, Massachusetts, and Ohio
Source: Prev Med Rep. 2025 Mar 24;53:103045. doi: 10.1016/j.pmedr.2025.103045 (PMC11995124; doi:10.1016/j.pmedr.2025.103045)
Supplement: Supplementary file 1 — Supplementary material [file mmc1.docx]

Supplemental Table 1: Business activity codes and description of providers in the US Drug Enforcement Administration database

| Code | Description |
| --- | --- |
| Business Activity Code C Sub Code 1 | Practitioner – DW/30 |
| Business Activity Code C Sub Code 4 | Practitioner – DW/100 |
| Business Activity Code C Sub Code B | Practitioner - DW/275 |
| Business Activity Code C Sub Code K | PRACTITIONER-DW/30SW |
|  |  |
| Business Activity Code M Sub Code F | MLP-Nurse Practitioner DW 30 |
| Business Activity Code M Sub Code H | MLP-Nurse Practitioner DW 100 |
| Business Activity Code M Sub Code K | MLP-Nurse Practitioner DW 275 |
| Business Activity Code M Sub Code Q | MLP-NURSE PRACTITIONER-DW/30SW |
|  |  |
| Business Activity Code M Sub Code G | MLP-Physician Assistant DW 30 |
| Business Activity Code M Sub Code I | MLP-Physician Assistant DW 100 |
| Business Activity Code M Sub Code L | MLP-Physician Assistant DW 275 |
| Business Activity Code M Sub Code R | MLP-PHYSICIAN ASSISTANT-DW/30SW |
|  |  |

Footnote: MLP: Mid-level practitioner, DW: Drug Addiction Treatment Act-waived
